# A re-evaluation of gender bias in receptiveness to scientific evidence of gender bias

**DOI:** 10.1098/rsos.240419

**Published:** 2024-09-04

**Authors:** David R. Shanks, Hollie A. Coles, Nadia Yeo

**Affiliations:** ^1^ Department of Experimental Psychology, University College London, 26 Bedford Way, London WC1H 0AP, UK

**Keywords:** bias, gender, scientific evidence

## Abstract

Gender bias has been documented in many aspects of Science, Technology, Engineering and Mathematics (STEM) careers, yet efforts to identify the underlying causes have been inconclusive. To what extent do cognitive biases, including unequal receptiveness in women and men to evidence of gender bias, contribute to gender bias in STEM? We investigated receptiveness in a STEM context among members of the general public, by undertaking a high-powered (total *N* = 1171) replication, including three experiments (2 pre-registered) of the prominent study by Handley *et al*. [22]. It was hypothesized that men would evaluate a research summary reporting evidence of gender bias less favourably than women but that there would be no difference between men and women’s evaluations of research summaries unrelated to gender bias. The results revealed no effect of the assessor’s gender on receptiveness to scientific evidence of gender bias. The different results compared to those of Handley *et al*. [22] suggest either that the gender bias they detected has diminished in the past decade or that their findings are a false positive. The present research adds to a growing body of evidence suggesting that some influential studies on cognitive ‘markers’ of gender bias warrant re-examination.

## 1. Introduction

Gender biases have been studied across a wide range of careers and employment sectors. Social and behavioral scientists have devoted considerable amounts of energy to studying the prevalence of gender bias within their own profession, and in particular Science, Technology, Engineering, and Maths (STEM). A high-level summary of the findings of several decades of research is that biases exist in many aspects of STEM (see [1], for a meta-analysis), but at the same time, there is robust evidence that most have been weakening in recent decades [2,3]. In a comprehensive review, Ceci *et al*. [3] summarized evidence relating to gender biases in key aspects of the academic career including faculty hiring, journal acceptances and salaries, and other work has examined citations (e.g. [4]) and grant applications (e.g. [5]). Ioannidis *et al*. [6] found, for instance, that among over nine million published authors, the male: female ratio declined from 3.93 prior to 1992 to 1.36 post−2011. Indeed, evidence suggests there is now a bias in the United States in favour of women for appointment to faculty positions in some disciplines [7] as well as the award of significant honours [8].

How important are cognitive biases in causing gender inequality? Causes of these gender gaps are probably numerous and include economic and institutional factors that impede women’s careers [9,10], gender differences in self-efficacy [11] and in interests and lifestyle preferences [12], as well as unequal impact of parenthood on productivity [13]. A smaller body of research has looked at potential individual-level cognitive underpinnings of gender bias. This research has catalogued a range of phenomena including explicit and implicit bias, stereotyping, and prejudice which are claimed to be instrumental in generating beliefs, attitudes, and behaviours that lead to women being treated unfairly compared to men.

A well-known example is the Implicit Association Test (IAT), which purports to assess unconscious bias. This test measures the valence of social categories indirectly via the speed of responding to congruent and incongruent category pairings. Although IAT studies [14] have found robust evidence of implicit gender stereotypes (e.g. males tending to be associated with science and females with arts), biases against women have not been found. In fact, IAT studies consistently report bias in favour of women [15,16]. In any case, the explanatory power of implicit sexist attitudes is weak because evidence shows that IAT sexism scores fail to correlate with real-world discrimination [5,17].

In another highly influential body of work, science faculty have been asked to evaluate identical application materials for job candidates with either male or female names. In the most highly cited study employing this method, Moss-Racusin *et al*. [18] found a robust bias against female applicants in terms of judged competence and hirability, and this bias was shown by both male and female faculty respondents. A high-powered registered replication project [19,20] has been undertaken to establish whether this pattern is still observed. In any event, the significance of the findings of Moss-Racusin *et al*. [18] is undermined by the fact that a meta-analysis [21] of many such studies shows that the bias weakens as the materials and experimental context are made more realistic, to the point where it is largely eliminated when experienced and motivated judges are provided with detailed and realistic applications from highly competent applicants (as would be the case in real job searches). Moreover, another more targeted meta-analysis (focusing only on STEM hirability) obtained an overall effect that was not significantly greater than zero [1].

Finding an individual-level cognitive bias is important as it would offer a target for piloting potential interventions before they are assessed in real-world situations. While the two bodies of research described above (IAT studies and job candidate evaluations) have thus far failed to identify a plausible cognitive factor underpinning gender bias, research on individual differences in receptiveness to evidence of gender bias appears more promising. Handley *et al*. [22] reported that relative to women, men tend to be more sceptical about the existence of gender bias, which they interpreted as a form of confirmation bias (although other explanations are possible, such as weaker expectations of gender bias in men). Their research provides a simple method for quantifying subtle and unintentional gender bias at the group level.

In the present article, we reconsider this method in detail and attempt to replicate Handley *et al*.’s key result. Across three experiments, Handley *et al*. [22] measured how receptive people are to experimental research reporting gender bias. Experiments 1 and 2 measured gender differences in participants’ evaluations of the overall quality of a journal article abstract (specifically, the abstract from Moss-Racusin *et al*. [18]) reporting gender bias. Participants were members of the general public (*N* = 205) in Experiment 1 and university faculty (*N* = 205) in Experiment 2. In both experiments, Handley *et al*. [22] reported that men evaluated research documenting gender bias less favorably than women. Experiment 3 was slightly different. It comprised a between-subjects design where a sample of the general population (*N* = 303) read either a journal abstract [23] reporting gender bias in STEM or an altered version of this abstract reporting no gender bias. The detailed results from all experiments are shown in the forest plot in figure 2, which is described in more detail later. The meta-analytic effect size across these experiments is a standardized mean difference (i.e. Cohen’s *d*) equal to 0.37, a small- to medium-sized effect. To put this bias in context, it is comparable in magnitude to other robust ‘textbook’ (e.g. [24]) cognitive biases such as sunk-cost [25] (*d* = 0.31) and framing [26] (*d* = 0.50) effects. We can convert the effect size into a number-needed-to-treat (NNT [27]); using the NNT function in the dmetar package in R [28]. If a male interviewer offered jobs to 7/14 interviewees, a female interviewer differing from the male one by 0.37 Cohen’s d units in her assessment standard would offer jobs to 8/14 (that is, to one of the 7 who would have been rejected by the male interviewer; NNT ≈ 7 for an event that occurs 50% of the time in the baseline condition). This is a consequential difference.

To elaborate on the concept of ‘bias’ in Handley *et al*.’s [22] research, we emphasize that just because men gave lower evaluations of the research than women does not mean that women are right and men wrong (or vice versa). Indeed to the extent that the evaluations are subjective, there is no yardstick against which their accuracy can be compared. It is the difference in men’s and women’s average ratings that constitutes evidence of bias, as no theory of evaluation with claims to be normative would include the judge’s gender as a valid cue. No inferences are being drawn about the magnitude of the bias within men or women.

Handley *et al*.’s results are important as they offer a simple laboratory method for quantifying gender bias. Indeed, the findings of Handley *et al*. [22] seem to be borne out by evidence [29] that research on gender bias is less likely to be funded or published than other research. This tendency could be a consequence of the bias identified in Handley *et al*.’s research with male editors and reviewers tending to be unreceptive to research on gender bias. Moreover, information absorbed from and the attitudes provoked by a brief research summary appear to be fairly consequential. Moss-Racusin *et al*. [30] presented participants with a news article reporting the evidence of Moss-Racusin *et al*. [18] documenting gender bias in STEM (gender bias condition), or the same article altered to make it appear that it found no evidence of gender bias (no gender bias condition). In the gender bias relative to the no gender bias condition, compared to men, women indicated significantly lower levels of connection to, positive attitudes towards, and desire to pursue STEM. In another study, Moss-Racusin, Molenda and Cramer [31] found further support for a lack of male receptiveness to gender bias. They conducted a non-experimental analysis of 831 comments written by the general public in response to three articles reporting the Moss-Racusin *et al*. [18] experimental evidence of gender bias against women. The researchers found that men were more likely on average than women to post negative responses while women were more likely on average than men to post positive ones.

Despite this promising evidence, the results of Handley *et al*. [22] merit further examination for at least two reasons. First, there has only been one (very recent) attempt to replicate these findings, a surprising fact given the high number of citations the work has garnered (over 300 Google Scholar citations as of March 2024) and the many studies that have replicated or extended the job application method of Moss-Racusin *et al*. [18]. Xiao *et al*. [32] conducted several pre-registered experiments in which participants rated the quality of the Moss-Racusin *et al*. [18] research. The research was described in a summary (which included a graphic of the results) rather than simply via the original article’s abstract, and in most experiments the evaluation questions were different from those employed by Handley *et al*. [22]. Xiao *et al*. [32] replicated the overall gender effect, with male participants giving lower ratings than females, in three experiments, whereas in two others the effect was not statistically significant. Even when the effect was replicated (for instance, in Experiment 1 which used the same evaluation questions as Handley *et al*.), it was appreciably smaller than that reported by Handley *et al*. as shown in figure 2. The meta-analytic effect size across these experiments is Cohen’s *d* = 0.22, around half the magnitude obtained across Handley *et al*.’s experiments. Xiao *et al*.’s main finding, however, was that participants’ moral commitment to gender equality (their perceptions of the issue as a moral imperative and as identity-defining) explained a considerable amount of variance in their evaluations of scientific research on gender bias, and indeed in those cases where participant gender was significantly associated with ratings, it no longer predicted evaluations when moral commitment to gender equality was included as a covariate.

A second reason to replicate Handley *et al*.’s research is that the results of their Experiment 3 are inconclusive. While they show a just-significant (*p* = 0.046) interaction between participant gender and abstract version (reporting evidence of gender bias or no gender bias), they failed to replicate the simple effect (that is, male evaluations being lower than female evaluations for the gender bias abstract) found in Experiments 1 and 2, yielding only a small and non-significant effect size (Cohen’s *d* = 0.20) with a Bayes factor (BF_10_ = 0.36) that actually favours the null to an ‘anecdotal’ degree. Thus, we do not know that the abstract used in Experiment 3 [23] is viewed differently by men and women.

Regarding laypeople’s (as opposed to faculty’s) receptivity to evidence of gender bias, Handley *et al*. [22] found that men judged such evidence more harshly than women in one experiment (Experiment 1) but not in another with a different abstract (Experiment 3). Moreover, they failed to demonstrate that this difference in receptiveness is specific to research summaries relating to gender bias, or that the difference generalizes from the specific Moss-Racusin *et al*. [18] abstract to a different one [23]. As such, the research conducted by Handley *et al*. [22] does not reveal whether men are uniquely harsh evaluators of scientific research pertaining to gender bias or are universally harsh evaluators of all scientific research.

Therefore, an important question is raised regarding whether a replication of this study (specifically, Handley *et al*. [22], Exp. 1), with the addition of a within-subjects control condition unrelated to gender bias, would yield the same results. This question forms the motivation for the present research.

## 2. Methods

We report how we determined our sample sizes, all data exclusions (if any), all manipulations and all measures in the study. Experiments 2 (https://osf.io/ej49t) and 3 (https://osf.io/wq9pe) were pre-registered. The experiments were run online as detailed below. For a review and discussion about the reliability of data collected online, see Rodd [33].

### 2.1. Design and hypotheses

All three experiments adopted the same mixed design in which article type (experimental/ control) was a within-subjects factor and participant gender (male/female) was a between-subjects factor. The dependent variable was the rating given to each abstract. The main hypotheses are that these factors will interact (H1) and specifically that male participants are selectively unreceptive (i.e. give lower ratings) to the experimental (reporting scientific evidence of gender bias) abstract (H2). As detailed below, the experiments differed in the specific neutral (control) abstract employed, and in Experiment 3, a set of comprehension questions was introduced.

Note that our focus (as in the research reviewed above) is on gender rather than sex, and participants were asked to report the former from a drop-down menu accompanying the request ‘Please choose your gender’, with the options male/female/non-binary/prefer not to say.

All studies were approved by the University College London (UCL) Department of Experimental Psychology ethics committee, and all participants gave informed consent.

### 2.2. Participants

For Experiment 1 a power analysis for an independent samples *t*‐test was conducted using G*Power [34]. Based on data from Experiment 1 of Handley *et al*. [22], the sample was calculated using an effect size of Cohen’s *d* = 0.45, alpha of 0.05 and power equal to 0.95. The projected sample size was 216. In total 259 participants recruited from Prolific (https://www.prolific.com/) completed the survey in exchange for £1. Data were excluded from one participant who responded ‘Prefer not to say’ when asked to report their gender, and from eight participants who failed the manipulation check.

This left a final total of 250 participants (121 male and 129 female), whose ages ranged from 18 to 71 ( M=32.10 years, s.d.=12.11 ). Of these, 214 (85.60%) were White, 12 (4.80%) Asian, 11 (4.40%) Black, 12 (4.80%) Other and 1 (0.40%) was undeclared.

The power analysis for Experiment 2 took a different approach (see pre-registration). It assumed an alpha of 0.05, power of 0.80, and a small effect size (*d* = 0.20). This effect size is based on the smallest effect in [22] (Exp. 3). Based on these parameters, the required sample size was 620 participants. A total of 633 individuals (314 men and 319 women), aged between 20 and 100 ( M=34.55 years, s.d.=11.64 ) completed the survey and provided usable data. Of these, 450 (71.09%) were White, 154 (24.33%) Asian, 21 (3.32%) Black, 3 (0.47%) Other and 1 (0.16%) was undeclared.

Consistent with the Handley *et al*. [22] procedure all participants were recruited via the online survey platform Amazon Mechanical Turk (MTurk; https://www.mturk.com). Participants were only eligible to complete the experiment if they met the following three conditions: first, if they were accredited with a minimum of a 95% human intelligence task (HIT) approval rate on MTurk (in order to enforce a higher quality of response); second, if they identified as either male or female; third, if they were aged 18 or over. Participants were paid $1.18 for taking part.

Experiment 3 was powered to detect an effect size likely to be of practical significance (i.e. *d* = 0.3), a power of 0.8, and an alpha of 0.05. 279 participants were recruited. Data were excluded from one participant whose gender was not provided.

The final sample comprised 137 men and 141 women from the United Kingdom (UK), aged 18 to 82 ( M=40.97 years, s.d.=14.11 ). Of these, 240 (86.33%) were White, 11 (3.96%) Asian, 13 (4.68%) Black, 5 (1.80%) Other and 1 (0.36%) was undeclared. Participants were recruited from Prolific and were compensated £2.25. The eligibility criteria to participate were that they must identify as either male or female and be older than 18.

Handley *et al*. [22] report mean ages of 30.12 (Experiment 1) and 34.22 (Experiment 3). The mean age in the present experiments is comparable, though slightly older in Experiment 3.

### 2.3. Materials

#### 2.3.1. Demographic questionnaire

This questionnaire asked participants to report their gender, age, highest level of education achieved, and ethnic group.

#### 2.3.2. Abstracts

Replicating Handley *et al*. [22], the experimental abstract in all experiments was ‘Science faculty’s subtle gender biases favour male students’ by Moss-Racusin *et al*. [18] (see appendix A). In Experiment 1 the control abstract was an edited version of Knobloch-Westerwick *et al*. [23] in which the text was altered to no longer report any evidence of gender bias (see appendix A). In Experiments 2 and 3, the control abstract was from Brehmer *et al*. [35]: ‘Working-memory training in younger and older adults: Training gains, transfer, and maintenance’ (see appendix A). This was a non-gender bias abstract closely matched to the experimental abstract in overall judged quality as determined in a pilot study (see electronic supplementary material). When shown to participants, all abstracts were accompanied by the article title and the first author’s family name. For some of their participants, Handley *et al*. [22] gave the first author a female first name and for others a male first name, and independently varied the institutional affiliation. Because these factors are unrelated to the main hypotheses and had inconsistent effects (see Handley *et al*. [22], Supporting Information, §5), we did not include them in the present experiments. In Experiment 1 we provided the first author’s affiliation together with the article’s keywords. In Experiments 2 and 3, the names and initials of all authors were provided as well as the journal name, publication year, volume, and page numbers (in Experiment 2 the DOI was also given).

In Experiment 3, a practice abstract was included, ‘Parents reading with their toddlers: The role of personalization in book engagement’ by Kucirkov *et al*. [36]. Additionally, there were three multiple-choice comprehension questions about each abstract. The questions were about the aim, a key method or procedure utilized, and the findings of the study. For example, the first question about the experimental abstract was ‘What is the aim of the study?’ The four response options were: ‘Compare men and women’s employment opportunities in the STEM field’, ‘Understand the influence of social media on gender discrimination’, ‘Investigate science faculty’s bias against female students that could contribute to the gender disparity in academic science’ and ‘Examine the impact of socioeconomic status and gender on academic achievement’. The correct answer was the third option. The response options were constructed to be sufficiently distinct and the correct option utilized the same phrasing as in the abstract. The pre-registered criterion to pass the comprehension check is to correctly answer at least two out of three questions about each abstract.

The questionnaire of Handley *et al*. [22] was used for rating abstracts in all experiments. A 6-point Likert scale (ranging from 1 (not at all) to 6 (very much)) was used to answer four questions, each related to a different aspect of the abstract: ‘To what extent do you agree with the interpretation of the research results?’, ‘To what extent are the findings of this research important?’, ‘To what extent was the abstract well written?’ and ‘Overall, my evaluation of this abstract is favourable.’

#### 2.3.3. Other questionnaires

In Experiment 1 participants also completed the collective self-esteem questionnaire [37] to measure their identification with their gender. The results are reported in the electronic supplementary material. Participants in Experiment 3 reported their beliefs about traditional gender roles using the five-item scale developed by Eccles [38]. Those (both male and female) who held stronger beliefs in traditional gender roles were less receptive to evidence of gender bias. The results are described in the electronic supplementary material.

### 2.4. Procedure

All studies were completed online via Qualtrics (https://www.qualtrics.com/uk/). After completing the consent form participants read an introductory page, detailing information about the task, including the following description from Handley *et al*. [22]: ‘In the scientific world, peer experts judge the quality of research and decide whether or not to publish it, fund it, or discard it. But what do everyday people think about these articles that get published? We are conducting an academic survey about people’s opinions about different types of research that was published back in the last few years. You will be asked to read two [Experiments 1 and 2] / three [Experiment 3] very brief research summaries and then answer a few questions about your judgments as non-experts about this research. There is no right or wrong answer and we realize you don’t have all the information or background. But just like in the scientific world, many judgments are made on whether something is quality science or not after just reading a short abstract summary. So to create that experience for you, we ask that you just provide your overall reaction as best you can even with the limited information. You will also be asked to provide demographic information about yourself’. Participants were also provided with UCL’s ethics committee approval number and confidentiality information. They then read and rated the experimental and control abstracts. Participants were instructed to: ‘Read the following abstract from a published research study, and then provide your opinion with the items below’.

In Experiment 1, the experimental abstract was always presented first on the basis that responses to this abstract would not be affected by responses to the control abstract. Participants answered the four quality rating questions after reading each abstract. In Experiments 2 and 3 the order was randomized.

In Experiment 3, a practice abstract [36] was read prior to the experimental and control abstracts. Participants were explicitly instructed to carefully read the abstract as there would be subsequent questions that tested their understanding. The aim of including the practice abstract was to emphasize to participants that the questions would be challenging and hence motivate them to study the subsequent abstracts carefully. After reading the practice abstract, they answered three comprehension questions. The procedure was similar for the experimental and control abstracts, except that prior to answering the comprehension questions, participants rated them for quality via the four questions detailed previously.

They then filled out the collective self-esteem questionnaire (Experiment 1) or the beliefs about traditional gender roles questionnaire (Experiment 3) before moving on to the demographic form. In Experiment 1 an attention check question was included. The comprehension check questions in Experiment 3 served as attention-check items. Finally, participants answered whether they had read any of the abstracts before taking part in the study and were debriefed.

### 2.5. Data analysis

We used R (Version 4.4.0 [39]) and the R-package *papaja* (Version 0.1.2 [40]) for all analyses. For all experiments, the four scale items demonstrated high internal consistency for both the experimental (Cronbach’s *α* = 0.83, 0.83 and 0.84, for Experiments 1–3, respectively) and control (Cronbach’s *α* = 0.85, 0.85 and 0.84, for Experiments 1–3, respectively) abstracts. Therefore, as in Handley *et al*.’s study, ratings were averaged to measure participants’ receptiveness to the quality of the research. The main analysis comprised a 2 × 2 mixed analysis of variance (ANOVA) on the ratings for each experiment with participant gender (male and female) as a between-subjects factor and abstract type (experimental, control) as a within-subjects factor. Subsequent tests examined whether gender influenced ratings for the experimental abstract separately.

## 3. Results

The amount of time taken to complete each part of the survey was not recorded. However the total completion time was M=481.88 sec, s.d.=387.21 , in Experiment 1, M=211.50 sec, s.d.=244.01 , in Experiment 2 and M=687.43 sec, s.d.=332.25 , in Experiment 3. The appreciably longer duration in Experiment 3 is consistent with the aim of including the comprehension questions, namely to increase engagement.

Experiments 2 and 3 proceeded according to their respective preregistration protocols.

### 3.1. Experiment 1

**Figure 1.**
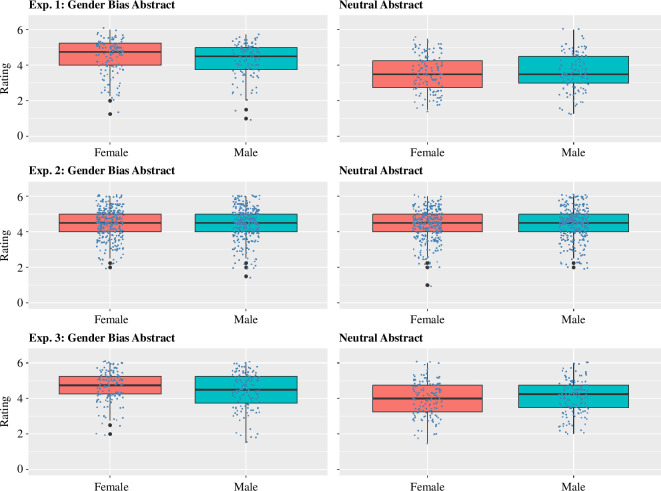
Ratings of the experimental and neutral (control) abstracts in Experiments 1–3. Dots are individual data points. The boxplots show the median and first and third quartiles.

The results are shown in the top two panels of figure 1. The ANOVA revealed no effect of Gender, F(1248)=0.42 , p=.518 , ${\hat{\eta }}_{G}^{2}=.001$ , 90% CI $\left[.000,.018\right]$ . In contrast, there was a main effect of condition, F(1248)=135.78 , p<.001 , ${\hat{\eta }}_{G}^{2}=.144$ , 90% CI $\left[.083,.212\right]$ , reflecting the fact that ratings were higher overall for the Experimental ( M=4.40 , s.d.=0.95 ) than the Control abstract ( M=3.59 , s.d.=1.02 ). The interaction was not significant, failing to support H1, F(1248)=2.50 , p=0.115 , ${\hat{\eta }}_{G}^{2}=.003$ , 90% CI $\left[.000,.025\right]$ . A one-tailed analysis (H2) focusing just on the Experimental abstract (males: M=4.31 , s.d.=0.92 , females: M=4.49 , s.d.=0.97 ) failed to reveal a significant effect of gender, ΔM=0.18 , 95% CI $\left[-0.02,\infty \right]$ , $t\left(247.97\right)=1.47$ , p=0.071 . A Bayesian *t*‐test shows that the data are more likely under the null than under H2 to an ‘anecdotal’ degree, ${BF}_{\text{10}}=0.38$ .

### 3.2. Experiment 2

The results are shown in the middle panels of figure 1. An equivalent ANOVA to that described above found no effect of gender, $F\left(1,631\right)=0.79$ , p=.374 , ${\hat{\eta }}_{G}^{2}=.001$ , 90% CI $\left[.000,.009\right]$ . Once again there was a main effect of condition, $F\left(1,631\right)=5.97$ , p=0.015 , ${\hat{\eta }}_{G}^{2}=.002$ , 90% CI $\left[.000,.011\right]$ , with ratings being slightly higher overall for the Experimental ( M=4.50 , s.d.=0.87 ) than the Control abstract ( M=4.43 , s.d.=0.88 ). Importantly, the interaction (H1) was not significant, $F\left(1,631\right)=0.36$ , p=0.547 , ${\hat{\eta }}_{G}^{2}=.000$ , 90% CI $\left[.000,.004\right]$ . A one-tailed analysis (H2) on ratings for the Experimental abstract (males: M=4.52 , s.d.=0.90 , females: M=4.48 , s.d.=0.85 ) found no effect of gender, ΔM=-0.04 , 95% CI $\left[-0.15,\infty \right]$ , $t\left(628.03\right)=-0.57$ , p=0.715 , and a Bayesian *t*‐test (not pre-registered) again shows that the data are more likely under the null than under H2 to a ‘substantial’, ${BF}_{\text{10}}=0.10$ .

### 3.3. Experiment 3

The results are shown in the bottom panels of figure 1. The ANOVA found no effect of Gender, F(1276)=0.25 , p=0.615 , ${\hat{\eta }}_{G}^{2}=.001$ , 90% CI $\left[.000,.014\right]$ . Once again there was a main effect of condition, F(1276)=69.32 , p<.001 , ${\hat{\eta }}_{G}^{2}=.072$ , 90% CI $\left[.031,.126\right]$ , with ratings being moderately higher overall for the Experimental ( M=4.55 , s.d.=0.94 ) than the Control abstract ( M=4.03 , s.d.=0.95 ). The interaction was not significant, F(1276)=3.60 , p=.059 , ${\hat{\eta }}_{G}^{2}=.004$ , 90% CI $\left[.000,.026\right]$ . A one-tailed analysis on ratings for the Experimental abstract (males: M=4.47 , s.d.=1.01 , females: M=4.64 , s.d.=0.87 ) found no effect of gender, ΔM=0.17 , 95% CI $\left[-0.02,\infty \right]$ , $t\left(267.25\right)=1.48$ , p=0.070 , and a Bayesian *t*‐test again shows that the data are more likely under the null than under H2 to an ‘anecdotal’ degree, ${BF}_{\text{10}}=0.38$ .

Participants answered 74.94% of the comprehension questions on the experimental and control abstracts correctly overall. A substantial number (84) of participants (30.22%) in Experiment 3 failed to correctly answer at least two out of the three comprehension questions for the experimental and control abstracts. This criterion was pre-registered to ensure that only participants who paid close attention to the abstracts would be included in the analysis. For the remaining 194 participants results revealed a significant difference between male ( M=4.56 , s.d.=0.98 ) and female ( M=4.77 , s.d.=0.79 ) mean receptiveness to the experimental abstract, ΔM=0.21 , 95% CI $\left[0.00,\infty \right]$ , $t\left(190.42\right)=1.65$ , p=0.050 , while the Bayesian analysis tended anecdotally to support the null hypothesis, ${BF}_{\text{10}}=0.54$ .

Because these results provide a hint that more attentive male participants are less receptive to evidence of gender bias in the experimental abstract compared to female participants, we undertook an additional exploratory analysis of data from all experiments. We took total survey completion time as a proxy for attentiveness, standardizing times within each experiment to eliminate irrelevant between-experiment differences in mean completion time. We then regressed the difference in overall quality ratings for the experimental and control abstracts onto participant gender and standardized completion time. The interaction in this regression tests the hypothesis that the more attentive participants are, the greater the male-female difference in quality ratings will be for the experimental compared to the control abstract. The relevant interaction term in the regression was however very small, b=0.05 , 95% CI $\left[-0.06,0.16\right]$ , and far from statistically significant, $t\left(1157\right)=0.97$ , p=0.333 . These and the comprehension check findings therefore fail to provide strong support for the hypothesis that the key finding will be more apparent amongst more attentive participants.

*Combined analysis*: Because the experiments had a common design we pooled all the data and ran another exploratory gender x condition ANOVA. This found no effect of gender, *F* (11159) = 0.03, p=.860 , ${\hat{\eta }}_{G}^{2}=.000$ , 90% CI $\left[.000,.002\right]$ . Once again there was a main effect of condition, *F* (11159) = 147.44, p<.001 , ${\hat{\eta }}_{G}^{2}=.031$ , 90% CI $\left[.016,.049\right]$ , with ratings being moderately higher overall for the Experimental ( M=4.49 , s.d.=0.91 ) than the Control abstract ( M=4.15 , s.d.=0.99 ). The interaction (H1) reached statistical significance, *F* (11159) = 5.35, p=.021 , ${\hat{\eta }}_{G}^{2}=.001$ , 90% CI $\left[.000,.007\right]$ , but with an extremely small effect size. A one-tailed analysis (H2) on ratings for the Experimental abstract (males: M=4.46 , s.d.=0.93 , females: M=4.52 , s.d.=0.88 ) found no effect of gender, ΔM=0.06 , 95% CI $\left[-0.03,\infty \right]$ , $t\left(1151.06\right)=1.05$ , p=0.147 , and a Bayesian *t*‐test again shows that the data are substantially more likely under the null than the alternative hypothesis, ${BF}_{\text{10}}=0.11$ .

To put the mean gender difference in ratings of the experimental abstract in context, it is equivalent to 1% on the 6-point rating scale—a tiny effect. The equivalent effect across Handley *et al*.’s Experiments 1–3 was about 6%.

## 4. Meta-analysis

**Figure 2.**
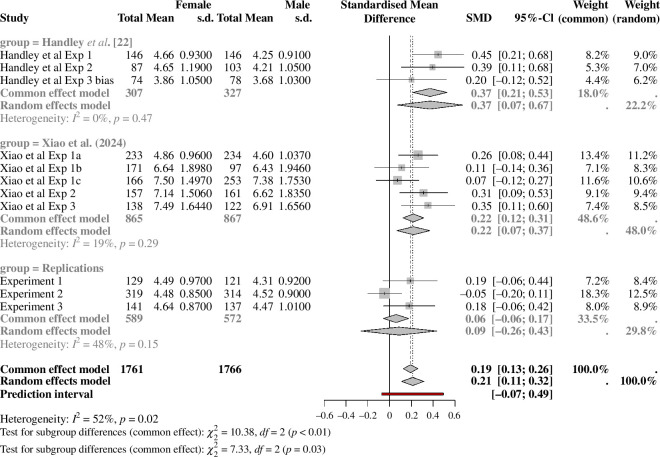
Forest plot of the data from Handley *et al*. [22], Xiao *et al*. [32], and the present experiments.

We conducted a non-preregistered meta-analysis using the *meta* package [41]. We report results for both fixed/common-effects and random-effects models. Figure 2 is a forest plot of the results. The top portion shows the effects from the three relevant studies by Handley *et al*. [22], the middle portion the effects from the five studies by Xiao *et al*. [32], and the bottom portion shows the effects from the three experiments described above. Although the overall effect size (fixed-effects standardized mean difference = 0.19, random-effects standardized mean difference = 0.21) across all studies is significantly different from zero, more important is that the meta-analysis finds a significant difference between the sets of studies (fixed-effects: $\chi^{2}$ = 10.38, p = 0.006; random-effects: $\chi^{2}$ = 7.33, p = 0.026). The pattern remains significant when the analysis excludes Xiao *et al*.’s experiments. Confirming the combined analysis reported above, the present replication experiments yield an effect size that is very small and whose confidence interval includes zero.

**Figure 3.**
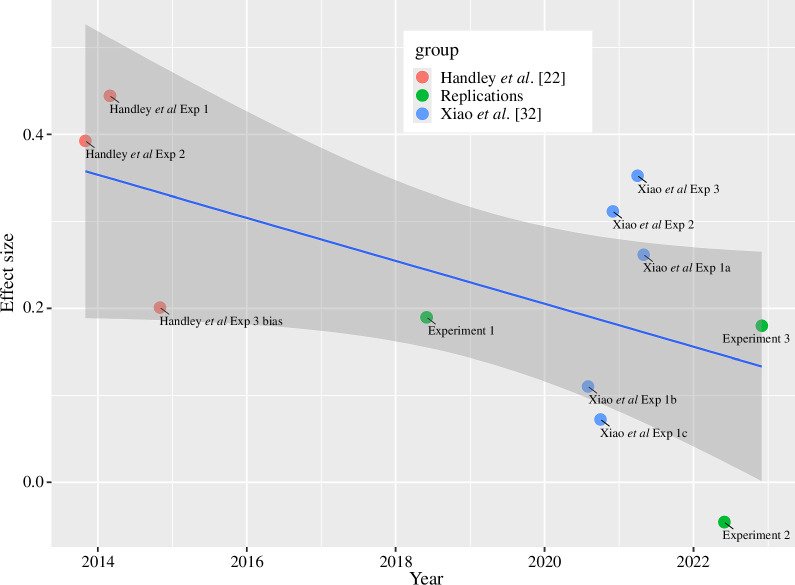
Effect sizes from Handley *et al*. [22], Xiao *et al*. [32] and the present experiments plotted against month/year of data collection.

The meta-analysis reveals another interesting pattern. As shown in figure 3, there is a suggestion that the effect is diminishing across time. Although the trend is not significant, $t\left(9\right)=-2.08$ , p=0.068 , this is unsurprising given the small number of observations. The expected effect now (i.e. in 2024) is very small (Cohen’s d ≈ 0.1) and, according to the regression, is predicted to reach zero in 2028. We discuss this further in §5.

## 5. Discussion

Confirmation bias is widespread in human judgement [42]. Handley *et al*. [22] reported a form of this bias whereby men, relative to women, tended to deprecate scientific evidence of gender bias presented in a short research summary. The present research however has not been able to reproduce this finding. Despite closely following Handley *et al*.’s methods—indeed despite improving on them in some respects (see below)—we failed to replicate their gender bias effect. Receptiveness ratings of male and female participants did not differ reliably.

In §1, we noted that the evidence reported by Handley *et al*. [22] was somewhat inconsistent. Focusing on non-faculty samples, one study (Experiment 1) obtained a statistically significant gender effect on the experimental (gender bias) abstract but another (Experiment 3) did not. We also noted that their results were ambiguous on the issue of demonstrating that the gender effect in a standard participant sample is larger on experimental than control (i.e. not reporting evidence of gender bias) abstracts. Their Experiment 1 did not include a control abstract. While their Experiment 3 did and found a just-significant participant gender by abstract type interaction, this was (as just noted) in the context of a nonsignificant gender effect on the experimental abstract. Handley *et al*. [22] did not, therefore, find evidence of a significant participant gender by abstract type interaction at the same time as a significant gender effect on the experimental abstract in either of these experiments.

In none of the three experiments reported here was there a significant interaction between abstract type (an experimental abstract reporting evidence of gender bias versus a neutral control abstract) and participant gender (H1). Focusing just on the experimental abstract, none of the experiments found a statistically significant rating difference between male and female participants (H2); in fact, in all of them, Bayesian analyses found stronger support for the null than the alternative hypothesis. When all the data were aggregated, the abstract type × gender interaction was statistically significant, but its effect size was barely greater than zero and again a Bayesian analysis supported the null hypothesis of no gender difference for the experimental abstract.

A meta-analysis reinforced and extended these conclusions. The overall effect obtained in Experiments 1–3 is very small (about 1% on the 6-point scale) and its confidence interval includes zero. Looking at the evidence as a whole, the meta-analytic effect is small (*d* ≈ 0.2) and 6 out of 11 experiments failed to detect an effect at *p* < 0.05. The meta-analysis furthermore finds that the effect obtained in the present experiments is significantly smaller than that reported by Handley *et al*. [22]. On the other hand, 10/11 experiments (including 6/7 pre-registered ones) found an effect numerically in the same direction as Handley *et al*. [22], with men having a systematic tendency, relative to women, to be unreceptive to and critical of scientific evidence of gender bias, as conveyed in a short text summary. Overall, it would be hard to deny that there is a true effect, but equally it is small. Despite a total sample of over 3500 participants, there appear still to be unknown moderators of this bias.

One potential moderator is the passage of time (figure 3). Handley *et al*.’s research was conducted nearly a decade ago and changes in societal perceptions of gender bias are entirely possible. Indeed, as noted in §1, there is widespread evidence of reductions in STEM gender bias in recent years [3]. While this may partly explain the smaller effects found here and in Xiao *et al*. [32], it does not explain the considerable heterogeneity found in the studies conducted between 2020 and 2022. It is unlikely that the samples who participated in the present experiments come from a different population than those tested by Handley *et al*. [22]. Recall that Experiment 2, in particular, sampled participants from Amazon MT with a similar age distribution to those tested by Handley *et al*. [22].

Another issue that must be considered is the possibility that the effects Handley *et al*. [22] obtained in their Experiments 1 and 2 were false positives, or at least yielded inflated effect sizes. We have already noted that their Experiment 3, in addition to the present experiments, failed to replicate the key effect of participant gender. Another aspect of their Experiments 1 and 2 is relevant to assessing the false-positive conjecture. Although their data analyses and discussion focused on the effect of participant gender, this was in fact only one of three independent variables in their design (but not that of Experiment 3). Handley *et al*. [22] gave the first author of the target abstract a female or male first name, and orthogonally associated the first author with either Yale University or Iowa State University. Interestingly, the results failed to conceptually replicate the effect reported by Knobloch-Westerwick *et al*. [23]; that is, quality ratings were not lower for female- compared to male-authored abstracts. But the key point is that these experiments included several factors each of which could have yielded a meaningful and reportable effect, increasing the probability that any one of them is a false positive [43].

It is of course important and appropriate to subject null results from replication studies to careful scrutiny [44]. The present experiments were well-powered and followed Handley *et al*.’s methods closely, but it is possible that consequential aspects of the experiments were different. An obvious issue is low participant engagement: can we be sure that our participants considered the abstracts sufficiently carefully for any real bias to be detectable? In fact there are at least two reasons to believe that they did. First, we know that the ratings of the quality of research we obtained were meaningful and not just random guesses: in the pilot study, different abstracts were rated very differently (see electronic supplementary material), we observed a consistent difference across Experiments 1–3 in ratings for the experimental and control abstracts, and in Experiment 3 ratings correlated with scores on the traditional gender roles questionnaire (see electronic supplementary material). Secondly, Experiment 3 specifically included a comprehension check and participants responded to a practice item and comprehension questions to ensure that they understood how challenging these questions would be. Their accuracy levels on these questions were high.

Here, we have not tested the claim that science faculty shows a gender bias. In their Experiment 2, Handley *et al*. [22] reported an overall receptiveness difference between male and female faculty, but also found that this effect was only shown by STEM but not non-STEM faculty. Extending the discussion elaborated below, it must be a possibility that the effect with STEM faculty was a false positive, and indeed an attempted replication provides some support for this [20]. In any event we deemed it a precondition of surveying a faculty sample that the effect should be initially replicated in a general participant sample. If it is difficult to replicate the gender bias effect that Handley *et al*. reported in a sample from an online survey platform, it seems unlikely that the interaction they reported amongst faculty (with STEM but not non-STEM faculty showing the effect) would be replicated. But future research might seek to acquire further evidence regarding this interaction.

Another limitation of the present experiments, as well as previous research on receptiveness to evidence of gender bias, is that it has focused almost entirely on summaries of a single piece of research, the Moss-Racusin *et al*. [18] study. The exceptionally high impact of this report (over 4000 Google Scholar citations as at June 2024) largely explains why Handley *et al*. [22] used it as the target abstract in their Experiments 1 and 2, why Moss-Racusin *et al*. [31] analysed 831 written comments made by members of the public in response to three news articles about the study, and why Moss-Racusin *et al*. [30] investigated men and women’s sense of belonging, positivity towards and aspirations to participate in STEM after reading a summary of the Moss-Racusin *et al*. [18] study or a version doctored to suggest that the experiment revealed no evidence of gender bias. This focus on Moss-Racusin *et al*.’s [18] study, and our aim specifically to replicate the study by Handley *et al*. [22], motivated us also to employ this abstract. As noted in §1, in their Experiment 3 Handley *et al*. [22] did not obtain a significant gender effect on ratings of a different abstract [23], but clearly it would be useful for future research to employ a wider set of summaries.

The present results cast doubt on the reproducibility and magnitude of one potential ‘marker’ of gender bias in STEM, namely receptivity to evidence of gender bias. In §1, we briefly reviewed other such surreptitious or indirect markers (e.g. the IAT) for which validity is also in doubt. It may therefore be the case that suitable methods for indirectly assessing gender bias will not be found and that researchers should focus on more traditional overt sexism scales, which tend to show good discriminant and predictive validity [17,45]. The present research also serves to emphasize the need for increased uptake of pre-registered studies and registered reports to ensure that the literature is more representative of the true extent of gender bias.

Businesses, universities and other institutions commit vast resources of money and staff time to unconscious bias training. However even proponents of the concept of unconscious bias admit the ‘disappointing conclusion’ that interventions to date ‘lack established methods that durably diminish implicit biases and have not reproducibly reduced discriminatory consequences of implicit (or other) biases’ [46, p. 7]. If subtle cognitive gender bias is so hard to demonstrate, as the present and other findings [21,47] suggest, then it is unsurprising that interventions designed to overcome this bias have proven so ineffective. We concur with Moss-Racusin *et al*. [30] that ‘if gender bias has no impact on men’s and women’s STEM outcomes, then current policies aimed at stamping out bias reflect wasted resources and missed opportunities’ (p. 651).

## Data Availability

This article was written in RMarkdown and papaja. The code for reproducing it, as well as the data, analysis scripts, and materials for all experiments are available at the Open Science Framework at [48]. The Stage 1 protocol can be viewed at [48]. Supplementary material is available online [49].

## Appendix A

### A.1. Abstracts

The Moss-Racusin abstract is the gender bias text in all 3 experiments. The Knobloch-Westerwick abstract was the control abstract in Experiment 1 and the Brehmer et al. abstract was the control abstract in Experiments 2 and 3.
